# STAT3 and Endothelial Cell—Cardiomyocyte Dialog in Cardiac Remodeling

**DOI:** 10.3389/fcvm.2019.00050

**Published:** 2019-04-24

**Authors:** Fouad A. Zouein, George W. Booz, Raffaele Altara

**Affiliations:** ^1^Department of Pharmacology and Toxicology, Faculty of Medicine, American University of Beirut, Beirut, Lebanon; ^2^Department of Pharmacology and Toxicology, School of Medicine, University of Mississippi Medical Center, Jackson, MS, United States; ^3^Department of Pathology, School of Medicine, University of Mississippi Medical Center, Jackson, MS, United States; ^4^Institute for Experimental Medical Research, Oslo University Hospital, University of Oslo, Oslo, Norway; ^5^KG Jebsen Center for Cardiac Research, Oslo, Norway

**Keywords:** signal transduction, transcription, cardiac hypertrophy, heart failure, angiogenesis, inflammation, oxidative stress

## Abstract

This article presents an overview of the central role of STAT3 in the crosstalk between endothelial cells and cardiac myocytes in the heart. Endothelial cell STAT3 has a key role in inflammation that underlies cardiovascular disease and impacts on cardiac structure and function. STAT3 in endothelial cells contributes to adverse cardiomyocyte genetic reprograming, for instance, during peripartum cardiomyopathy. Conversely, cardiomyocyte STAT3 is important for maintaining endothelial cell function and capillary integrity with aging and hypertension. In addition, STAT3 serves as a sentinel for stress in the heart. Recent evidence has revealed that the redox nature of STAT3 is regulated, and STAT3 is responsive to oxidative stress (ischemia-reperfusion) so as to induce protective genes. At the level of the mitochondrion, STAT3 is important in regulating reactive oxygen species (ROS) formation, metabolism, and mitochondrial integrity. STAT3 may also control calcium release from the ER so as to limit its subsequent uptake by mitochondria and the induction of cell death. Under normal conditions, some STAT3 localizes to intercalated discs of cardiomyocytes and serves to transmit pro-fibrotic gene induction signals in the nucleus with increased blood pressure. Further research is needed to understand how the sentinel role of STAT3 in both endothelial cells and cardiomyocytes is integrated in order to coordinate the response of the heart to both physiological and pathological demands.

## Introduction

Signal transducer and activator of transcription 3 (STAT3) was first recognized around 1994 as the acute-phase response factor that is activated by inflammation and couples to enhanced gene expression (1). Of the 7 mammalian STAT transcription factors, only loss of the STAT3 gene is embryonically lethal (2). STAT3 is activated by a number of growth factors and cytokines, and plays a key role in cell growth, differentiation, and survival. Studies in the past 2 decades have shown that STAT3 is important in protecting the heart under stress conditions, including ischemia, reperfusion, increased blood pressure, and with overt failure. The reader is referred to other recent reviews for an in-depth coverage of STAT3 signaling in the heart (1, 3).

**Figure 1:**
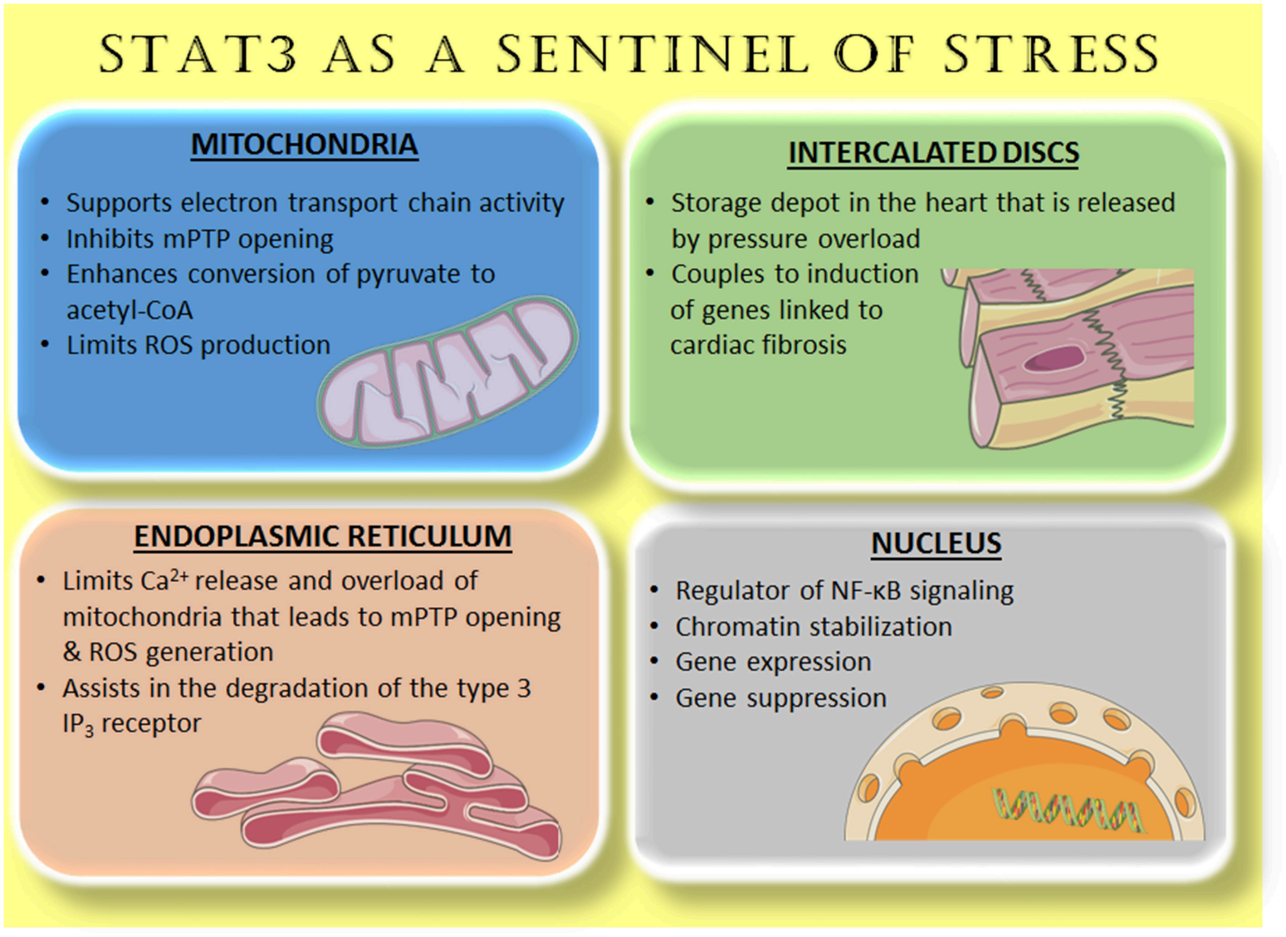
STAT3 as a sentinel of stress. Accumulating evidence reveals that STAT3 is located within various cellular compartments, or relocates there with stress stimuli. In this capacity, STAT3 serves to maximize efficient respiration, limit ROS generation, and prevent apoptosis with ischemia-reperfusion (mitochondria and endoplasmic reticulum). In the nucleus, constitutive, and enhanced STAT3 modulates cytokine and inflammatory signaling. A pool of STAT3 constitutively associated with β_IV_-spectrin at intercalated discs responds to increased wall stress on the heart by remodeling of the extracellular matrix through gene induction. See text and previous reviews (1, 3) for additional details. Images are from Servier Medical Art (https://smart.servier.com/).

This brief review is focused on the central role of STAT3 in the crosstalk between endothelial cells and cardiac myocytes, and is part of the Research Topic, *Cardiac Microvascular Endothelium Contribution to Cardiac Myocyte Growth, Structure, and Contractile Function*. Endothelial cell STAT3 plays a key role in inflammation that underlies cardiovascular disease and contributes to adverse cardiomyocyte genetic reprograming during peripartum cardiomyopathy. In addition, recent evidence suggests that endothelial STAT3 may indirectly contribute to cardiomyocyte metabolism and function via its effects on endothelial autophagy. On the other hand, cardiomyocyte STAT3 is important for maintaining endothelial cell function and capillary integrity. We begin with an overview of the increasingly appreciated role of STAT3 as a sentinel for stress in the heart. Accumulating evidence indicates that STAT3 is both redox-sensitive and positioned within endothelial cells and cardiac myocytes at various sites to respond quickly to outside stress stimuli (Figure 1).

## STAT3 as a Sentinel of Stress

Two sites of phosphorylation important for canonical STAT3 activation are located in a COOH-terminal transcription activation domain (TAD) (1). Y705 phosphorylation leads to homodimer formation, which is followed by translocation to the nucleus and gene induction. Phosphorylation of S727 boosts the transcriptional activity of STAT3 through the recruitment of transcriptional cofactors, for instance p300/CREB-binding protein (CBP). Although STAT3 functions as a critical transcription factor, a new paradigm has emerged for its role as a sentinel and communicator of cellular stress. Several aspects of this novel task are discussed here.

Ischemia or ischemia–reperfusion (IR) is reported to induce the translocation of STAT3 to mitochondria in cardiac myocytes by undefined means (4, 5), which may involve phosphorylation of S727 within the TAD. This translocation has been associated with cardiac protection through modulation of reactive oxygen species (ROS) production and prevention of the mitochondrial permeability transition pore (mPTP) opening (3). STAT3 acetylation was recently implicated in its translocation to mitochondria in serum-starved cells after serum re-introduction or insulin stimulation (6). In this case, the presence of STAT3 in mitochondria enhanced pyruvate metabolism and ATP synthesis.

STAT3 has been shown to modulate mitochondrial complex activity and ROS production by several means (1, 3). STAT3 may interact with the structural subunit of complex 1 GRIM19 or NDUFA13 to directly modulate the electron transport system and reduce ROS formation with IR (7). Multiple studies have reported that STAT3 is required for the maximal activity of several mitochondrial complexes, most consistently complexes I and II (8). Mitochondrial STAT3 was found to associate with pyruvate dehydrogenase complex E1 (PDC-E1), thereby enhancing conversion of pyruvate to acetyl-CoA, the mitochondrial membrane potential, and ATP synthesis (6). By interacting with cyclophilin D, STAT3 inhibits opening of the mPTP, thereby preserving mitochondrial integrity and limiting ROS generation (9, 10). Although a direct role of STAT3 in regulating mitochondrial ROS in cardiac myocytes has been questioned because of insufficient levels of STAT3 protein (11), that possibility would be less problematic in endothelial cells, as these cells have a much lower number of mitochondria. Finally, STAT3 protects mitochondria indirectly as a transcription factor, for example, by upregulating the anti-apoptotic gene Bcl2 or SOD2 (12).

STAT3 contains multiple cysteine residues that are redox-sensitive (3). Recent evidence indicates that their oxidation under conditions of increased ROS is a regulated process involving peroxiredoxin-2. In HEK293T cells stimulated with interleukin-6 (IL-6) or oncostatin M, STAT3 associated with peroxiredoxin-2, which resulted in the oxidation of multiple cysteine residues of STAT3, and formation of higher order STAT3 complexes and attenuated gene expression (13). In mouse hearts with cardiac myocytes that produced increased levels of H_2_O_2_ due to a deficiency in a mitochondrial complex I protein, peroxiredoxin-2 expression was elevated and was associated with an increase in STAT3 dimerization and Bcl2 gene expression (12). These hearts were more resistant to ischemia-reperfusion (IR) injury due to enhanced expression of the STAT3 target and antiapoptotic protein, Bcl2.

Recent evidence revealed that STAT3 localizes to the endoplasmic reticulum (ER) of certain cancer cells so as to limit Ca^2+^ release and subsequent Ca^2+^ overload of mitochondria that leads to opening of the mPTP and ROS generation (14, 15). This anti-apoptotic action of STAT3 was attributable to its association with and subsequent degradation of the type 3 IP_3_ receptor, a Ca^2+^-release channel. The degradation process was dependent upon STAT3 S727 phosphorylation. Whether a similar scenario occurs in cardiac endothelial cells or cardiomyocytes awaits investigation. Besides a direct action at the level of the ER, STAT3 has been linked to protective effects against ER stress during moderate hypoxia of cardiac myocytes by enhancing the unfolded protein response (UPR). This occurs via STAT3-mediated suppression of miR-199a-5p levels and the subsequent upregulation in expression of the UPR-related proteins activating transcription factor 6 (ATF6) and 78 kDa glucose-regulated protein (GRP78) (16).

In cardiac myocytes, a pool of STAT3 was recently shown to be associated with the cytoskeletal protein β_IV_-spectrin at intercalated discs (17). Activation of Ca^2+^/calmodulin-dependent kinase II (CaMKII), with transverse aortic constriction so as to mimic hypertension, caused phosphorylation of β_IV_-spectrin and the displacement of STAT3. Once displaced, STAT3 translocated to the nucleus and was implicated in the induction of genes for fibrosis and loss of cardiac function, but not cardiac hypertrophy. Thus, STAT3 may serve as a sensor in the heart to assuage increased wall stress through increased extracellular matrix deposition. However, the possible role of STAT3 at intercalated discs in regulating genes of cardiac myocytes that are important for endothelial cell function, such as vascular endothelial growth factor (VEGF), was not reported.

## Endothelium to Cardiomyocyte Communication

A growing body of evidence has shown that inflammation underlies cardiovascular disease and remodeling, with the endothelium playing a key role in initiating and sustaining an inflammatory response (18–25). STAT3 contributes to endothelial inflammation at many levels. It has been linked to the induction of NADPH oxidases (26, 27), and may be activated by oxidative stress (28). Additionally, STAT3 in endothelial cells couples to the expression of cytokines, such as IL-6 and chemokine (C-C motif) ligand 2 (CCL2) (29, 30), as well as cell adhesion molecules, e.g., fractalkine (*aka* CX3CL1), vascular cell adhesion molecule 1 (VCAM1), and intercellular adhesion molecule 1 (ICAM1) (31, 32). It is reported that endothelial tyrosine kinase Bmx with the subsequent activation of STAT3 is needed for angiotensin II-induced cardiac hypertrophy and fibrosis due to induction of pro-inflammatory cytokines, IL-6 and IL-8 (33).

On the other hand, STAT3 may exert anti-inflammatory actions involving endothelial cells. STAT3 may suppress iNOS levels by binding to its promoter, and thereby have anti-oxidant and anti-inflammatory properties (34). In endothelial cells, STAT3 is reported to be involved in the production of vasodilators prostacyclin and nitric oxide (NO), via the upregulation of eNOS, and is implicated in endothelial cell proliferation and survival, as well as pro-angiogenic/survival signaling by VEGF and erythropoietin (EPO) (35–39). In addition, STAT3 has anti-inflammatory and anti-apoptotic actions through the expression of mitochondrial manganese-superoxide dismutase (SOD2) and Bcl-xl (3).

Endothelial cell STAT3 has a protective role in post-ischemic myocardial function. In a mouse model of IR injury, ablation of endothelial cell STAT3 worsened capillary integrity and cardiac function, and increased myocardial inflammatory signaling (40). Greater cardiomyocyte expression of IL-6 and apoptosis was observed.

In addition to driving cardiac hypertrophy and fibrosis through the upregulation of IL-6 and IL-8 (33), endothelial STAT3 may help to regulate autophagy in endothelial cells and thereby affect cardiac myocyte metabolism and function. In this way, endothelial STAT3 may contribute to the impact of starvation or obesity on the cardiac remodeling that occurs with hypertension or ischemia. For instance, disruption of endothelial autophagy was recently shown to reduce fatty acid storage in the heart and its reliance on fatty acid oxidation (41). This was attributed to reduced expression of lipid chaperone proteins in endothelial cells that are important for the shuttling of fatty acids across the endothelium into cardiac myocytes. In addition, endothelial cell-specific deletion of the leptin receptor, which couples prominently to STAT3 activation, was shown by others to enhance endothelial autophagy with pressure overload of the mouse heart (42). This was associated with improved angiogenesis, as well as reduced hypertrophy and fibrosis (42). The authors suggested that a blunted upregulation of endogenous autophagy inhibitors, including STAT3, may have explained these results. However, the role of STAT3 in autophagy is complex (43), and additional studies are needed to understand its role in endothelial cell autophagy in various disease contexts.

Taken together, evidence indicates that endothelial STAT3 can have either a beneficial or detrimental impact on cardiac myocytes depending upon pathophysiological context. Further research is needed to understand whether these actions are mediated by specific sentinel compartments for STAT3 in endothelial cells.

## Cardiomyocyte to Endothelium Communication

**Figure 2:**
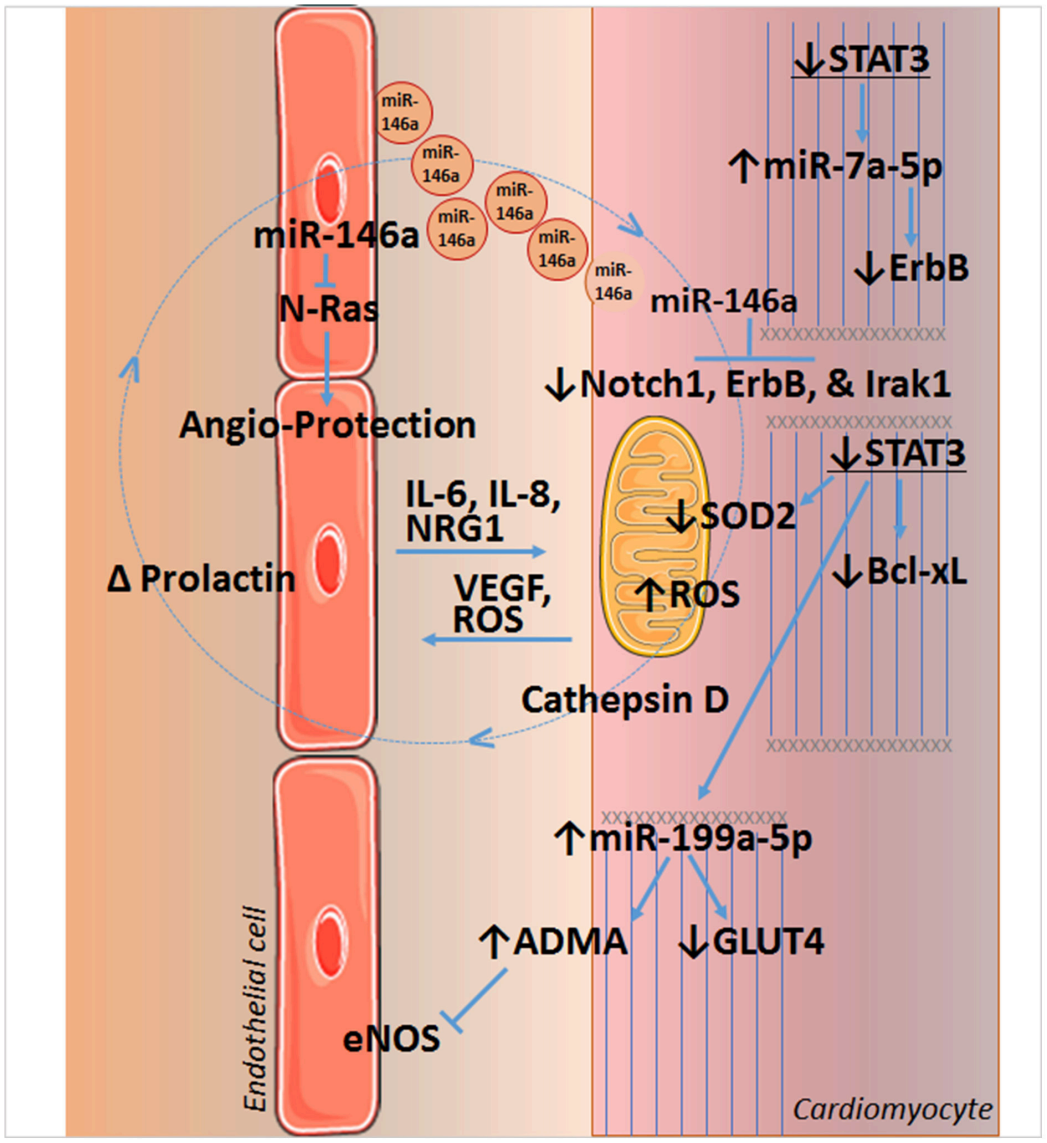
STAT3 and crosstalk between endothelial cells and cardiomyocytes of the heart. Loss of STAT3 in cardiomyocytes leads to a decrease in SOD2 and Bcl-xL expression, resulting in increased reactive oxygen species (ROS) formation and apoptosis, respectively. Increased ROS is implicated in the upregulation of cathepsin D and formation of a shortened version of prolactin in peripartum cardiomyopathy, which induces miR-146a in endothelial cells. miR-146a has angiotoxic actions by targeting *NRAS*, which encodes for N-Ras that may act downstream of the VEGF or EGF receptors. Released in endosomes, endothelial miR-146a is taken up by cardiomyocytes and downregulates Notch1 (with beneficial or harmful effects that are context dependent), inflammatory Irak1, and the cytoprotective neuregulin receptor ErbB. Loss of cardiomyocyte STAT3 also upregulates miR-7a-5p that targets ErbB, as well as miR-199a-5p. Upregulation of miR-199a-5p decreases expression of the glucose transporter GLUT4 and leads to an increase in the endogenous inhibitor of eNOS and endothelial function, asymmetric dimethylarginine (ADMA), by impeding the ubiquitin-proteasome system (UPS). Impairment of UPS is associated with disruption of sarcomere structure as well. Cardiomyocyte STAT3 is also linked to VEGF expression. See text for additional details. Images of endothelial cells and mitochondrion are from Servier Medical Art (https://smart.servier.com/).

The expression of STAT3 in cardiac myocytes is required for myocardial capillary growth postnatally. In the mouse heart, targeted knockout of STAT3 in cardiomyocytes with the Cre-Lox system was associated with a gradual reduction in capillary density after birth that becomes significant at 2–3 months (44). Previously, others had reported that constitutive activation of STAT3 in cardiomyocytes leads to increased capillary density in the mouse heart due to enhanced secretion of VEGF (45). However, expression levels of critical pro-angiogenic proteins, HIF-1α, VEGF, and bFGF were not altered by STAT3 KO, although evidence for the release of an inhibitory paracrine factor was found. A subsequent study revealed a rather complex scenario in which STAT3 in cardiac myocytes regulates the ubiquitin-proteasome system (UPS) via suppression of miR-199a-5p expression by non-canonical means (45) (Figure 2). STAT3 KO was thus associated with an impaired UPS due to reduced levels of ubiquitin-conjugating enzymes Ube2g1 and Ube2i, which together with the resultant increased levels of protein arginine methyltransferase I (PRMT-I) caused increased synthesis of the endothelial cell and eNOS inhibitor, asymmetric dimethylarginine (ADMA).

Upregulation of miR-199a-5p with STAT3 KO, along with an increase in miR-7a-5p, sensitizes the normal and peripartum heart to chronic β-adrenergic signaling with isoproterenol (46). Cardiomyocytes rely on glucose oxidation in this case, as isoproterenol depletes serum free fatty acids, and reduces cardiac free fatty acid uptake and triglycerides (46, 47). However, increased miRNA-199a-5p suppresses glucose transporter-4 (GLUT4) levels, and miR-7a-5p suppresses levels of the cardioprotective receptor for neuregulin 1 (NRG1), ErbB. Moreover, impaired glucose uptake and metabolism leads to oxidative stress and increased mitochondrial ROS formation, as there is insufficient NADPH generated to maintain adequate levels of the antioxidant, GSH in mitochondria. It should be noted NRG1 is produced by cardiac microvascular endothelium and is important for the structural and functional integrity of the heart and angiogenesis (Figure 2) (48–51).

Endothelial cell STAT3 has a key role in inflammation that underlies cardiovascular disease and impacts on cardiac structure, function, and genetic reprograming; conversely, cardiomyocyte STAT3 is important for maintaining endothelial cell function and capillary integrity. The condition peripartum cardiomyopathy (PPCM) illustrates several aspects of the interplay between endothelium and cardiomyocyte communication, including its derangement. This life-threatening pregnancy-associated cardiomyopathy occurs in previously healthy women. Of note, STAT3 protein levels in the left ventricle are decreased in patients with end-stage heart failure due to PPCM (52). In pregnant female mice, a reduction in cardiac myocyte STAT3 predisposes the heart to adverse remodeling (52). In part, this occurs due to an increase in ROS formation due to reduced expression of the mitochondrial anti-oxidant protein SOD2, a direct gene target of STAT3 (53) (Figure 2). Increased oxidative stress in turn leads to increased cathepsin D expression and activity by cardiac myocytes, which forms an anti-angiogenic and pro-apoptotic cleaved form of the nursing hormone prolactin. The shortened prolactin acts on endothelial cells to increase miR-146a expression, which has angiotoxic effects via suppression of *NRAS* (54). In addition, miR-146a that is released in exosomes and taken up by cardiac myocytes, impairs the metabolic activity of cardiac myocytes and reduces their expression of Notch1, ErbB4, and Irak1 (53, 54). Moreover, enhanced Akt activity in PPCM due to prolactin or interferon-gamma (IFN-γ), further worsens redox balance and SOD2 loss, due to the down-regulation of anti-oxidative transcription factor FoxO3A and activation of p66SHC (53). Akt activation also drives cardiac inflammation through induction of the pro-inflammatory chemokine CCL2, which recruits macrophages. In addition to cardiac STAT3 protein levels being decreased in PPCM patients with end-stage heart failure (52), PPCM patients also exhibit elevated serum levels of cathepsin D and cleaved prolactin (53).

## Conclusions and Future Directions

STAT3 serves a critical function on coordinating crosstalk between cardiomyocytes and endothelial cells of the heart (Table 1). This two-way communication may be beneficial as exemplified by postnatal growth of capillaries, which in turn ensures adequate nutrient and oxygen delivery to the myocardium. Although a contributor in some aspects of inflammation, endothelial cell STAT3 also acts to protect the myocardium from IR, in part by limiting inflammatory cytokine expression in cardiomyocytes. Independent of any mitochondrial actions, STAT3 in cardiomyocytes is important for maintaining their metabolic homeostasis (46, 47, 55), and whether the same is true of endothelial cells of the heart will need to be determined. Additionally, the role that mitochondrial STAT3 of endothelial cells has in regulating cardiac function and remodeling will need to be studied. Although strong evidence has shown that STAT3 distributes to subcellular localizations to serve as a sentinel under stress conditions, further research is needed to understand how this role in both endothelial cells and cardiomyocytes is integrated in order to coordinate the response of the heart to both physiological and pathological demands.

**Table 1:** Contribution of cell-specific STAT3 to endothelial-cardiomyocyte crosstalk.

**Endothelial STAT3**	**Effect on cardiomyocytes**
KO	With IR, greater IL-6 expression, apoptosis, and worsened function
IL-6 and IL-8 gene expression	Enhanced hypertrophy and fibrosis with pressure overload
Suppression of autophagy	Hypertrophy, fibrosis, and impaired angiogenesis with pressure overload
**Cardiomyocyte STAT3**	**Effect on endothelial cells**
Overexpression-induced VEGF	Increased capillary density
KO	Release of ADMA or ROS resulting in reduced capillary growth and endothelial dysfunction with maturation or aging

*ADMA, asymmetric dimethylarginine; IL-6, interleukin 6; IL-8, interleukin 8; IR, ischemia-reperfusion; KO, knockout; ROS, reactive oxygen species. VEGF, vascular endothelial growth factor*.

## Author Contributions

All authors listed have made a substantial, direct and intellectual contribution to the work, and approved it for publication.

### Conflict of Interest Statement

The authors declare that the research was conducted in the absence of any commercial or financial relationships that could be construed as a potential conflict of interest.

## Abbreviations

ADMA: asymmetric dimethylarginine
ATF6: activating transcription factor 6
Bcl-2: B-cell lymphoma 2
Bcl-xL: B-cell lymphoma-extra large
bFGF: basic fibroblast growth factor
Bmx: bone marrow kinase in chromosome X
CaMKII: Ca^2+^/calmodulin-dependent kinase II
CBP: CREB-binding protein
CCL2: chemokine (C-C motif) ligand 2
CREB: cAMP response element-binding protein
CX3CL1: chemokine (C-X3-C motif) ligand 1
eNOS: endothelial nitric oxide synthase
EPO: erythropoietin
ErbB: epidermal growth factor receptor
FOXO3A: forkhead box O3A
GLUT4: glucose transporter-4
GRIM19: gene associated with retinoic-interferon-induced mortality 19
GRP78: 78 kDa glucose-regulated protein
GSH: glutathione
HIF-1α: hypoxia-inducible factor 1-alpha
ICAM1: intercellular adhesion molecule 1
IFN-γ: interferon-gamma
IL-6: interleukin-6
IL-8: interleukin-8
iNOS: inducible nitric oxide synthase
IP_3_: inositol 1,4,5-trisphosphate
Irak1: interleukin-1 receptor-associated kinase 1
mPTP: mitochondrial permeability transition pore
NDUFA13: NADH dehydrogenase [ubiquinone] 1 alpha subcomplex subunit 13
NO: nitric oxide
*NRAS*: neuroblastoma RAS viral oncogene homolog
NRG1: neuregulin 1
p66SHC: 66 KDa isoform of Shc
PDC-E1: pyruvate dehydrogenase complex E1
PRMT-I: protein arginine methyltransferase I
ROS: reactive oxygen species
SOD2: manganese-superoxide dismutase or superoxide dismutase 2
Ube2g1: ubiquitin-conjugating enzyme E2 G1
Ube2i: ubiquitin conjugating enzyme E2I
VCAM1: vascular cell adhesion molecule 1
VEGF: vascular endothelial growth factor.
